# The Role of Vitamin D in Non-Scarring Alopecia

**DOI:** 10.3390/ijms18122653

**Published:** 2017-12-07

**Authors:** Agnieszka Gerkowicz, Katarzyna Chyl-Surdacka, Dorota Krasowska, Grażyna Chodorowska

**Affiliations:** Chair and Department of Dermatology, Venereology and Paediatric Dermatology, Medical University of Lublin, Radziwiłłowska 13, 20-080 Lublin, Poland; kasiachyl@gmail.com (K.C.-S.); dorota.krasowska@umlub.pl (D.K.); grazyna.chodorowska@umlub.pl (G.C.)

**Keywords:** vitamin D, vitamin D receptor, alopecia

## Abstract

Non-scarring hair loss is a common problem that affects both male and female patients. Since any disturbances in the hair follicle cycle may lead to hair shedding, or alopecia, it is not surprising that the possible role of vitamin D in alopecia was investigated in many studies. Vitamin D has been shown to have many important functions. A growing body of evidence shows that vitamin D and its receptor are responsible for maintaining not only calcium homeostasis but also skin homeostasis. Moreover, vitamin D could also regulate cutaneous innate and adaptive immunity. This paper presents a review of current literature considering the role of vitamin D in alopecia areata, telogen effluvium, and female pattern hair loss. The majority of studies revealed decreased serum 25-hydroxyvitamin D levels in patients with different types of non-scarring alopecia, which could suggest its potential role in the pathogenesis of hair loss. According to the authors, vitamin D supplementation could be a therapeutic option for patients with alopecia areata, female pattern hair loss, or telogen effluvium. However, further studies on a larger group of patients are required.

## 1. Introduction

Vitamin D plays an important role in human health. Its main source is photosynthesis in the skin, whereas lower amounts are derived from nutrition and diet supplements [1]. The role of vitamin D in regulation of calcium homeostasis is well established. It stimulates the intestinal absorption of calcium and phosphate, reduces their renal clearance, and promotes bone mineralization [2,3,4]. Apart from calcium homeostasis, vitamin D has also been shown to modulate both innate and adaptive immunity [5]. It stimulates differentiation of monocytes into classical macrophages and enhances the chemotactic and phagocytic capacity of macrophages [6]. Wang et al. [7] demonstrated that 1,25-dihydroxyvitamin D (1,25(OH)_2_D) activates the transcription of antimicrobial peptides such as cathelicidin antimicrobial peptide in isolated human keratinocytes, monocytes, and neutrophils and enhances expression of defensin β2 in primary cultures of adult keratinocytes. Moreover, they observed that 1,25(OH)_2_D alone or in conjunction with lipopolysaccharide (LPS) induced cathelicidin antimicrobial peptide expression and release of antimicrobial activity in neutrophils. It has been proposed that induction of antimicrobial peptide expression by 1,25(OH)_2_D could be involved in the suppressive effects of ultraviolet B (UVB) radiation on innate immunity and suggested the potential of its analogues in treatment of opportunistic infections [7]. Furthermore, 1,25(OH)_2_D has been found to suppress antigen presentation by dendritic cells, [8] and to modulate the function of T helper (Th) lymphocytes by suppressing the synthesis of T helper type 1 (Th1) proinflammatory cytokines and enhancing cytokine production by T helper type 2 (Th2) cells [9]. 1,25(OH)_2_D has also been shown to suppress the T helper type 17 (Th17) related cytokine expression [10]. Besides Th lymphocytes, 1,25(OH)_2_D also induces apoptosis in activated B cells, suppresses B-cell proliferation, and decreases plasma cell and class-switched memory B-cell generation [11].

Since the immunomodulatory effect of vitamin D was demonstrated, its potential role has been studied in many areas of medicine. A growing body of evidence shows that vitamin D and its receptor may be involved in skin homeostasis [1,4,12,13,14,15,16].

Skin is a target organ for vitamin D not only because this compound is mostly synthesized in the epidermal keratinocytes and dermal fibroblasts from 7-dehydrocholesterol upon exposure to UVB radiation, but also because skin is able to respond to the active metabolites of vitamin D [1]. As mentioned above, vitamin D synthesis begins in the skin. The previtamin D is subsequently converted by a spontaneous thermal isomerization into vitamin D, which after bonding to a carrier protein is transported to the liver and hydroxylated to 25-hydroxyvitamin D (25(OH)D). The serum level of this form is a marker of vitamin D status. However, the biologically active form 1,25-dihydroxyvitamin D (1,25(OH)_2_D) is formed in the kidneys [1].

It was demonstrated that keratinocytes are capable of metabolizing vitamin D to the active form because they possess the activity of 1-α-hydroxylase and 25-hydroxylase [17]. Other cells including macrophages and dendritic cells are also capable of synthesizing the active form of vitamin D, and this process is predominantly regulated by immune signals, but not by calcium or parathyroid hormone concentration [18]. This is important because many of those cells are involved in the pathogenesis of different skin diseases [12,18,19].

Vitamin D exerts its action through the vitamin D receptor (VDR) [4,18,19,20,21]. VDR is expressed in keratinocytes, dendritic cells, macrophages, B and T lymphocytes, and in two major cell populations in the hair follicle: epidermal keratinocytes and mesodermal dermal papilla cells [12,19,22]. VDR is crucial for hair follicle integrity [22]. Its expression is required for normal hair follicle cycling but not for morphogenesis, and its deficiency can inhibit keratinocytes differentiation and disturb the normal postnatal hair follicle cycle [12,17,19,23]. It was demonstrated that patients with type IIA vitamin D-dependent rickets (VDDR IIA) develop alopecia between 1–3 months of age together with osteomalacia, dental caries, hyperparathyroidism, and mineral disturbances, including hypocalcaemia and hypophosphatemia [23,24,25]. Histopatological studies of patients with VDDR IIA demonstrated abnormalities of the hair follicles, including the presence of dermal cysts and irregular epidermal structures in the lower part of the hair follicle [25]. It was demonstrated that in VDR null mice, the hair follicles in catagen become dystrophic and the dermal papilla separates from the rest of the hair follicle as catagen progresses; consequently, anagen is not reinitiated [26]. Therefore, it has been proposed that VDR is required for anagen initiation [1]. Histologic changes within the hair follicle similar to those described in VDDR IIA were observed in atrichia with papular lesions (APL), characterized by total alopecia and papular and milia like growths that developed after birth [25]. Hence, it has been suggested that the *VDR* gene and *Hairless* gene (*Hr*) may be involved in the same genetic pathway that regulates postnatal hair cycling [23,25,27]. Moreover, it was found that VDR deficiency leads to increased *Hr* expression [26].

Since the role of VDR in the hair follicle cycle was confirmed, the potential role of vitamin D was the focus of many studies. It has been demonstrated that VDR knockout mice have increased levels of the active form of vitamin D. However, the possible toxic effect of 1,25-dihydroxyvitamin D on hair follicles was not proven [28]. Recently, Mady et al. demonstrated a possible role of calcium and 1,25(OH)_2_D in the regulation of postnatal hair follicle cycling [29]. They found that calbidin-D_9k_ knockout pups born of calbidin-D_9k_ knockout females fed with a low-calcium and vitamin D-deficient diet developed transient alopecia during the first postnatal catagen. However, alopecia was not present when these pups were suckled by mothers fed with a diet containing calcium and vitamin D [29].

Considering that any disturbances in the hair follicle cycle may lead to hair loss, it is not surprising that the possible role of vitamin D in alopecia was investigated in many studies. The aim of this paper is to review the current literature considering the role of vitamin D in non-scarring alopecia.

## 2. Vitamin D in Alopecia Areata

Alopecia areata (AA) is a common form of hair loss, characterized by sharply demarcated, round to oval, skin-coloured patches of non-scarring alopecia [30,31]. Study results support the autoimmune nature of AA, including: association with other autoimmune diseases, presence of hair follicle-specific autoantibodies, or improvement after immunosuppressive treatment [30,31,32]. Typical histological features of AA are: inflammatory infiltrate around and within hair follicles, composed mainly of Th1 cells and increased expression of interleukin-2 (IL-2) and interferon γ (IFNγ) [30,31,32]. At present, studies concentrate on finding the mediators involved in the pathogenic processes in AA, which could be a target for new therapeutic options [30,31]. 

Literature data suggest that vitamin D, due to its immunomodulatory effect, may be involved in the pathogenesis of AA [12,16,33].

Decreased serum 25(OH)D levels in AA in comparison to healthy subjects were reported in many studies [12,13,16,33,34,35,36,37], (Table 1).

Mahamid et al. suggested that serum 25(OH)D levels < 30 ng/mL were associated with AA occurrence [16]. D’Ovidio et al. reported that decreased serum 25(OH)D levels were accompanied by a compensatory increase of parathyroid hormone levels, which confirmed the real deficiency of vitamin D in patients with AA [13].

Interestingly, data concerning the correlation between serum 25(OH)D levels and clinical disease parameters are inconsistent. Yilmaz et al. did not find any correlation between 1,25(OH)_2_D or 25(OH)D concentrations in 42 patients with AA and the extent of hair loss, number of patches, disease duration, and nail involvement [33]. This was confirmed in another study [36]. In contrast, Aksu Cerman et al. demonstrated for the first time that serum 25(OH)D levels were inversely correlated with severity of AA assessed by Severity of Alopecia Tool (SALT) score [12]. This correlation was observed in further studies [34,35,37]. It has been suggested that lower serum levels of 25(OH)D found in more severe stages of AA might result from emotional stress due to severe hair loss which might discourage patients from public appearance and therefore from sun exposure [12]. However, it cannot be excluded that the observed differences between studies might result from different enrolment criteria, including AA severity and an unequal number of examined patients [13,33,35,36,37]. Moreover, another possible explanation could be the seasonal variation in serum 25(OH)D levels. Aksu Cerman et al. conducted a study during winter, whereas Yilmaz et al. during summer [12,33]. In addition, data regarding serum vitamin D levels among female and male patients with AA are also inconclusive. There are studies that point to lower levels in male patients than in females [13,36], studies that present opposite results [12,37] and studies that demonstrate no correlation with gender [34]. This can be explained by different cultural and religious aspects that influence vitamin D synthesis [12,33,34,36].

Since the majority of studies were conducted on adult patients, the study by Unal et al., is of special interest. The authors demonstrated vitamin D deficiency in paediatric patients with AA and in the control group. However, they reported significant inverse correlation between serum 25(OH)D levels and disease severity, duration, as well as the number of bald patches [40]. The authors suggested that vitamin D deficiency may aggravate the disease and lead to severe hair loss [40].

The potential role of VDR in the pathogenesis of AA was also assessed. Fawzi et al. revealed significantly lower levels of tissue and serum VDR in AA than in a control group. An important negative correlation was observed between the extent of AA and tissue VDR [47]. The results are in accordance with the study by Lim et al. [48]. The authors found significantly lower expression of VDR in hair follicles and epidermis within alopecia lesions than in healthy skin. Moreover, the VDR levels were lower in patients with a more severe form of hair loss. Reduction of VDR expression in AA was related to decreased hair cycle-related signals—Wnt/β-catenin signals. The authors suggested that reduced expression of VDR in AA might be related primarily to suppression of cell differentiation, since decreased expression of involucrin and filaggrin within hair follicles and epidermis were revealed [48].

Despite the fact that many studies reported deficiency or insufficiency of vitamin D in patients with AA, few reports evaluate the topical application of vitamin D analogues as a therapeutic option. Therefore, data reported by Kim et al., appear interesting [19]. The authors observed complete hair regrowth in the affected area after application of a topical calcipotriol solution (50 μg/mL) for 3 months in a 7-year old boy. During a 6-month follow-up, no hair loss was observed. A skin biopsy performed before treatment revealed loss of VDR expression in affected hair follicles. VDR expression was detected again after hair regrowth [19]. The results of these studies seem to confirm the correlation between expression of VDR and clinical hair regrowth, and highlight the role of vitamin D in AA.

Later, the effectiveness of topical application of 0.005% calcipotriol was evaluated in 22 adult patients with AA and scalp involvement lower than 40%. After three months of therapy, 59.1% of patients had hair regrowth; no response was observed in 36.4% of patients and worsening in 0.04%. A better clinical effect was observed in patients with a single bald patch. Among all patients, 91% had vitamin D deficiency. Interestingly, faster hair regrowth was observed in patients with lower serum 25(OH)D levels [49]. Similarly, improvement of mild-moderate AA after topical application of calcipotriol cream was also reported [50].

To sum up, the observed decreased serum 25(OH)D levels in AA patients suggest its role in the disease pathogenesis. It was demonstrated that vitamin D shifts the Th1 immune response toward Th2. Since AA is mainly Th1-mediated, the vitamin D deficiency may be involved in the development of hair loss [31,35]. The main limitation of the studies are small study groups, different inclusion criteria, and collection of samples during different seasons. 

## 3. Vitamin D in Female Pattern Hair Loss

Female pattern hair loss (FPHL) is one of the most common types of alopecia in women. Clinically, it is characterized by diffuse hair shedding with maintained frontal hairline [51]. Recent literature data include genetic, hormonal, and environmental factors in the pathogenesis of FPHL [52]. The possible link between serum 25-hydroxyvitamin D and FPHL has been suggested since its decreased concentration was demonstrated in patients with FPHL compared to control group [41,42,43] (Table 1).

Moneib et al. reported a significantly lower serum 25(OH)D levels in patients with FPHL than in controls [43]. The majority of patients with FPHL (96.6%) showed a vitamin D deficiency or insufficiency. Sufficient levels were observed only in 3.3% of patients. There was no significant difference between different serum 25(OH)D levels and mean disease duration or patients’ age; however, a significant difference between the severity of hair loss and mean serum 25(OH)D concentration was observed. The authors suggested that the higher serum 25(OH)D concentration in patients with most severe hair loss in comparison with less severe alopecia may result from increased exposure to ultraviolet light due to more decreased scalp hair density [43]. Contrary to this, in another study, patients with mild and moderate FPHL had significantly higher mean serum levels of 25(OH)D compared to those suffering from the severe form [41]. It cannot be excluded that conflicting results observed in both studies were determined by different patterns of sun exposure and evaluation of serum 25(OH)D level in different parts of the year.

It has also been pointed out that women with positive family history of FPHL and vitamin D deficiency or insufficiency are more prone to develop FPHL in comparison with women with sufficient serum 25(OH)D levels [43]. Contrary to this, Banihashemi et al. did not find any significant correlation between serum 25(OH)D concentration and positive family history of FPHL [42].

Although a decreased serum 25(OH)D concentration was demonstrated in FPHL, the role of VDR in FPHL was assessed in a single study. Fawzi et al. [47] reported significantly decreased concentrations of both serum and tissue VDR in androgenic alopecia in comparison with healthy controls. No correlation was observed between serum or tissue VDR concentration and disease severity. Interestingly, female patients had higher levels of both serum and tissue VDR than male patients [47]. This could be explained by the interaction of 17β-estradiol and 1,25(OH)_2_D, which results in enhancement of *VDR* gene expression [53].

At present, there are no studies evaluating the oral supplementation of vitamin D or its topical application in patients with FPHL.

The results described above suggest a possible involvement of vitamin D in pathogenesis of FPHL. However, the studies were limited by the small number of enrolled patients and different inclusion criteria. To elucidate the role of vitamin D in FPHL, further large-scale studies are required [41].

## 4. Vitamin D in Telogen Effluvium

Telogen effluvium (TE) is a common clinical problem defined as a non-scarring, diffuse hair loss that usually occurs 3 months after exposure to a triggering factor and is usually self-limiting. Causative factors considered in the pathogenesis include: stress, febrile states, drugs, endocrine abnormalities, and nutritional disturbances [54].

In a study conducted by Rasheed et al., the authors compared serum 25(OH)D levels in female patients with chronic TE, FPHL, and healthy controls. They revealed significantly lower serum 25(OH)D levels in comparison to the control group. The lowest level was observed in patients with the most severe hair loss. According to the authors, the results may indicate that vitamin D is involved in TE [41]. Similar results were reported by Nayak et al. [44]. Interestingly, the levels were significantly lower in female patients when compared with the females in the control group, whereas the difference between serum 25(OH)D concentrations in male cases and controls was not significant [44]. Contrary to these studies, Karadag et al. found significantly higher serum 25(OH)D levels in patients with TE than in the control group [45]. Moreover, serum 25(OH)D concentration in the higher quadrant was associated with a higher risk of developing TE. According to the authors, the observed increased serum 25(OH)D levels in TE might not be the cause, but rather a compensatory effect to the hair loss [45]. Loss of melanin synthesis in telogen hair follicles may result in increased vitamin D synthesis in the skin [55]. It was suggested that screening for vitamin D might be beneficial in the management of TE; however, data on the effects of vitamin D supplementation in hair loss is lacking.

Due to the limited number of studies and different methodologies it is difficult to compare the results. However, since nutritional disturbances are one of the triggering factors causing hair loss, the possible role of vitamin D or its deficiency should be studied further.

## 5. Conclusions

The studies revealed important alterations in serum 25(OH)D levels in patients with different types of non-scarring alopecia. In the majority of reports, the mean serum levels of 25(OH)D were decreased when compared to the healthy control, which might suggest a causal role of this decrease in the pathogenesis of hair loss. However it cannot be excluded that observed low serum 25(OH)D levels are secondary to the disease. Emotional stress related to severe hair loss might discourage patients form going outdoors as much as those without this condition. Therefore their potential for vitamin D synthesis might be reduced. Despite different mechanisms of AA, FPHL, and TE, it seems a reasonable therapeutic strategy to monitor serum 25(OH)D levels and introduce vitamin D supplementation in case of its deficiency or insufficiency in patients with AA, TE, and FPHL. It is worth noting that due to different methods used to evaluate the serum concentration of 25(OH)D, caution should be warranted when comparing the results. Other limitations of the studies include unequal study groups, different inclusion criteria, and the possible influence of seasonal variability of serum 25(OH)D concentration, since the samples were collected during different seasons. Therefore, there is an urgent need to standardize the laboratory measurement of vitamin D status that would be accurate, comparable over time, localization, or laboratory [56,57]. Further studies including larger groups of patients with standardized inclusion criteria and standardized protocols of 25(OH)D measurement are required to evaluate the vitamin D status and the effect of vitamin D supplementation in alopecia.

## Figures and Tables

**Table 1:** Clinical studies evaluating serum concentration of 25(OH)D in non-scarring alopecia.

Authors	Study Subjects (Number and Age)	Severity of Alopecia	Serum Concentration of Vitamin D (25(OH)D)		
			Criteria for Vitamin D Status	Method of Serum 25(OH)D Measurement	Notable Findings
Aksu Cerman et al., 2014 [12]	AA—86 patients V—44 patients C—58 healthy controls Mean age of: AA—32.21 ± 9.60 years V—33.64 ± 11.51 years C—32.55 ± 9.78 years	S1—71 patients S2—15 patients	D ≤ 20 ng/mL^−1^	LC-MS/MS	Significantly higher prevalence of vitamin D deficiency in AA than in V and C (*p* = 0.003, *p* < 0.001 respectively). A significant negative correlation between AA severity and serum concentration of 25(OH)D (*p* < 0.001 *r* = −0.409).
D’Ovidio R et al., 2013 [13]	AA—156 patients C—148 healthy controls Mean age of: AA—37.8 years C—34.5 years	AA multilocularis—49 patients Ophiasis—69 patients AT-AU—38 patients Minimal hair loss in all groups: 25% of scalp area	D < 20 ng/mL	CHL	Presence of serum 25(OH)D levels < 20 ng/mL significantly higher in AA vs. C (*p* < 0.025). In AA higher compensatory levels of PTH (*r* = −0.24, *p* < 0.01);
Mahamid et al., 2014 [16]	AA—23 patients C—20 healthy controls Mean age of: AA—24.2 ± 12.3 years C—27 ± 11.26 years	Patchy AA—18 Extensive AA—5	S—30–50 ng/mL I < 30 ng/mL D < 20 ng/mL	EIA	Serum 25(OH)D concentration significantly decreased in AA vs. C (*p* < 0.05); Serum 25(OH)D levels < 30ng/mL and CRP > 1 associated with AA occurrence (*p* = 0.02, *p* = 0.04, respectively).
Yilmaz et al., 2012 [33]	AA—42 patients C—42 healthy controls Mean age of: AA—30.8 ± 8.2 years C—29.3 ± 7.4 years	S1—30 patients S2—6 patients S3—3 patients S4—2 patients S5—1 patient	25(OH)D—insufficient concentration < 50 nmol/L 1,25(OH)_2_D—decreased concentrations ≤ 30 pg/mL	ELISA	Significantly lower concentration of 25(OH)D and 1,25(OH)_2_D in AA vs. C (*p* < 0.001, *p* < 0.001 respectively). No correlation between the levels of 25(OH)D and 1,25(OH)_2_D and disease severity, duration, nail involvement.
Bakry et al., 2016 [34]	AA—60 patients C—60 healthy controls Mean age of: AA—20.70 ± 10.85 years C—23.71 ± 7.45 years	Mild—24 patients Moderate—20 patients Severe—16 patients	S > 75 nmol/L I—50–75 nmol/L D < 50 nmol/L	ELISA	Significantly lower levels of serum 25(OH)D in AA vs. C (*p* < 0.001). Significantly lower serum levels of 25(OH)D in severe AA vs. moderate and mild (*p* = 0.03, *p* = 0.002 respectively).
Ghafoor et al., 2017 [35]	AA—30 patients C—30 healthy controls Mean age of: AA—23.77 ± 8.86 years C—24.03 ± 8.62 years	S1—4 patients S2—7 patients S3—12 patients S4—1 patient S5—6 patients	S—30 ng/dL I—20–29ng/dL D < 20 ng/dL	EIA	Significantly lower serum 25(OH)D levels in AA vs. C (*p* = 0.001). Lower serum 25(OH)D levels in patients with higher SALT Score.
Darwish et al., 2017 [36]	AA—30 patients C—20 healthy controls Mean age of: AA—28.67 ± 10 years C—24.8 ± 6 years	S1 (mild)—10 patients S2 (moderate)—7 patients S3–S5 (severe)—13 patients	NA	ELISA	Significant decrease of serum 25(OH)D concentration in AA vs. C (*p* < 0.001). In AA significantly lower serum 25(OH)D level in males vs. females (*p* = 0.009). No correlation with SALT score.
Attawa et al., 2016 [37]	AA—23 patients C—23 healthy controls Mean age of: AA—26.44 ± 10.87 years C—29.39 ± 8.10 years	S1—14 patients S2—3 patients S3–S5—6 patients	S > 30 ng/mL I—10–30 ng/mL D < 10 ng/mL	ELISA	Significantly lower serum 25(OH)D levels in AA vs. C (*p* = 0.01). Significant difference between vitamin D status and AA severity (*p* = 0.02).
Erpolat et al., 2017 [38]	AA—41 patients C—32 healthy controls Mean age of: AA cases—32.8 ± 7.5 years C—32.7 ± 7.5 years	Single patch—15 patients Multiple patches—26 patients	S > 30 ng/mL I—20–30 ng/mL D < 20 ng/mL	HPLC	No significant difference in serum 25(OH) D levels between AA and control (*p* > 0.05). Vitamin D deficiency—93.8% patients with AA
Bhat et al., 2017 [39]	AA—50 patients C—35 healthy controls Mean age of: AA cases—22.4 ± 8.6 years C—29.2 ± 7.6 years	S1—38 patients S2*—12 patients	D < 30ng/mL	CHL	Serum 25(OH)D levels significantly lower in AA vs. C (*p* < 0.001). A significant negative correlation between SALT score and serum vitamin D levels (*p* < 0.001; *r* = −0.730).
Unal et al., 2017 [40]	AA—20 paediatric patients C—34 paediatric healthy controls Mean age of: AA M/F—12.4 ± 4.2/13.3 ± 4.4 years C—M/F 16.6 ± 0.8/16.5 ± 1.01 years	S1—6 patients S2—9 patients S3—5 patients	D ≤ 20ng/mL	NA	Vitamin D deficiency in both groups with no significant differences between the groups (*p* = 0.084). Significant inverse correlation between serum 25(OH)D levels and SALT score, disease duration and number of patches (*p* < 0.001, *r* = −0.831, *p* < 0.001, *r* = −0.997, *p* < 0.001 *r* = −0.989 respectively ).
Rasheed et al., 2012 [41]	TE—42 patients FPHL—38 patients C—40 healthy controls Mean age of: TE and FPHL—29.8 ± 9.3 years C—30.8 ± 8.56 years	TE: Mild—22 patients Moderate—6 patients Severe—14 patients FPHL: Mild—15 patients Moderate—13 patients Severe—10 patients	S > 75 nmol/L I—25–75 nmol/L D < 25 nmol/L	Competitive enzyme immunoassay	Significantly lower serum 25(OH)D levels in TE and FPHL vs. C (*p* < 0.001). The highest serum 25(OH)D levels in mild vs. severe FPHL and TE (*p* = 0.035, *p* = 0.203 respectively).
Banihashemi et al., 2016 [42]	FPHL—45 patients; C—45 healthy controls Mean age of: FPHL—29.11 ± 7.31 years C—28.82 ± 7.11 years	Ludwig I—28 patients Ludwig II—2 patients Ludwig III—2 patients	S > 30 ng/mL I—20–30 ng/mL D < 20 ng/mL	ELISA	Lower serum 25(OH)D levels in FPHL vs. C (*p* = 0.04). No significant correlation between serum 25(OH)D levels and duration or severity of FPHL (*p* = 0.77, *p* = 0.92 respectively).
Moneib et al., 2014 [43]	FPHL—60 patients C—60 healthy controls Mean age of: FPHL—26.4 ± 4.51 years C—25.85 ± 4.49 years	Ludwig I—34 patients Ludwig II—22 patients Ludwig III—4 patients	S > 30 ng/mL I—21–29 ng/mL D < 20 ng/mL IN > 150ng/mL	RIA	Significantly lower mean serum 25(OH)D level in FPHL vs. C (*p* = 0.0001). Significant difference between serum 25(OH)D levels and Ludwig’s three degrees (*p* = 0.006). The highest serum 25(OH)D levels in Ludwig III.
Nayak et al., 2016 [44]	Diffuse hair loss—22 patients C—22 healthy controls Mean age of the study population—20.89 years	NA	I—25–75 nmol/L D < 20–25 nmol/L	ELISA	Significantly lower serum 25(OH)D levels among cases vs. C (*p* = 0.007).
Karadag et al., 2011 [45]	TE—63 patients C—50 healthy controls Mean age of: TE—29.0 ± 11.9 years C—28.4 ± 9.4 years	Acute TE—29 patients Chronic TE—34 patients	NA	RIA	Significantly higher serum 25(OH)D levels in TE patients vs. C (*p* < 0.01). Significantly increased risk for TE for patients with 25(OH)D levels in the highest quadrant vs. the lowest one (*p* < 0.0001).

AA—alopecia areata; AT—alopecia totalis; AU—alopecia universalis; TE—telogen effluvium; FPHL—female pattern hair loss; V—vitiligo; C—healthy controls; NA—not applicable; D—vitamin D deficiency; I—vitamin D insufficiency; S—vitamin D sufficiency; IN—vitamin D intoxication; S1—<25% scalp hair loss; S2—25–49% scalp hair loss; S2*—26–50% scalp hair loss; S3—50–74% scalp hair loss; S4—75–99% scalp hair loss; S5—100% scalp hair loss; SALT—Severity of Alopecia Tool; F—female; M—male; PTH—parathyroid hormone; 25(OH)D—25-hydroxyvitamin D; 1,25(OH)_2_D—1,25 dihydroxyvitamin D; CHL—chemilumiscence; EIA—enzyme immunoassay; ELISA—enzyme-linked immunosorbent assay; HPLC—high performance liquid chromatography; RIA—radioimmunoassay; LC-MS/MS—liquid chromatography/tandem mass spectrometry. Factor to convert units of 25(OH)D from ng/mL into nmol/L: 1 ng/mL = 0.400641 nmol/L [46].
